# Involvement of butyrate in electrogenic K^+^ secretion in rat rectal colon

**DOI:** 10.1007/s00424-018-2208-y

**Published:** 2018-09-25

**Authors:** Akihiro Inagaki, Mikio Hayashi, Naaz Andharia, Hiroko Matsuda

**Affiliations:** 1 Medical Research Project, Institute of Biomedical Sciences, Tokushima University Graduate School, 3-18-15 Kuramoto, Tokushima, 770-8503 Japan; 2 Department of Physiology, Kansai Medical University, 2-5-1 Shinmachi, Hirakata, 573-1010 Japan

**Keywords:** Short-chain fatty acid, Rectal colon, Short-circuit current, KCNQ

## Abstract

Short-chain fatty acids (SCFAs), such as acetate, propionate, and butyrate, are synthesized from dietary carbohydrates by colonic bacterial fermentation. These SCFAs supply energy, suppress cancer, and affect ion transport. However, their roles in ion transport and regulation in the intracellular environment remain unknown. In order to elucidate the roles of SCFAs, we measured short-circuit currents (I_SC_) and performed RT-PCR and immunohistochemical analyses of ion transporters in rat rectal colon. The application of 30 mM butyrate shifted I_SC_ in a negative direction, but did not attenuate the activity of epithelial Na^+^ channels (ENaC). The application of bumetanide, a Na^+^-K^+^-2Cl^−^ cotransporter inhibitor, to the basolateral side reduced the negative I_SC_ shift induced by butyrate. The application of XE991, a KCNQ-type K^+^ channel inhibitor, to the apical side decreased the I_SC_ shift induced by butyrate in a dose-dependent manner. The I_SC_ shift was independent of HCO_3_^−^ and insensitive to ibuprofen, an SMCT1 inhibitor. The mucosa from rat rectal colon expressed mRNAs of H^+^-coupled monocarboxylate transporters (MCT1, MCT4, and MCT5, also referred to as SLC16A1, SLC16A3, and SLC16A4, respectively). RT-PCR and immunofluorescence analyses demonstrated that KCNQ2 and KCNQ4 localized to the apical membrane of surface cells in rat rectal colon. These results indicate that butyrate, which may be transported by H^+^-coupled monocarboxylate transporters, activates K^+^ secretion through KCNQ-type K^+^ channels on the apical membrane in rat rectal colon. KCNQ-type K^+^ channels may play a role in intestinal secretion and defense mechanisms in the gastrointestinal tract.

## Introduction

Short-chain fatty acids (SCFAs: mainly acetate, propionate, and butyrate) are produced by anaerobic bacterial fermentation from dietary fiber within the lumen of the mammalian colon [51]. SCFAs are considered to play important roles as a source of nutrients and suppressant of colonic carcinoma [21]. They are transported into cells through the apical membrane by ionic transport pathways [45, 47] and non-ionic diffusion [10]. SCFA transport is regarded as an important factor that regulates colonic fluid balance, absorption of NaCl, and luminal and intracellular pH [40]. The pKa of SCFAs is approximately 4.8 and, thus, most SCFAs are present as anions at physiological pH 6–7 in the lumen of the colon [18, 51]. The carrier-mediated transport of ionized SCFAs into colonic epithelial cells includes (1) Na^+^-coupled transporters for monocarboxylates (SMCT1, also referred to as SLC5A8), (2) SCFA^−^/HCO_3_^−^ exchangers (primarily SLC26A3 with the possible additional involvement of SLC26A6), and (3) H^+^-coupled monocarboxylate transporters (MCT1, MCT4, and MCT5, also referred to as SLC16A1, SLC16A3, and SLC16A4, respectively) [51].

Frank et al. [19] previously reported that SMCT1 was expressed on the apical side, but not on the basolateral membrane of the colonic mucosa of mice. SMCT1 has been shown to possess the capacity for electrogenic SCFA transport with a 3:1 [13] or 2:1 [14, 23] stoichiometry between Na^+^ and monocarboxylates. Electroneutral SCFA^−^/HCO_3_^−^ exchange is regarded as the main transport pathway for ionized SCFAs in the intestine and colon [40, 45]. Electroneutral MCTs, MCT1 and members of its family, were previously reported to affect electrogenic transport [7]. The localization of MCT1 to the small and large intestines of humans, cows, and rodents remains controversial [19, 22, 38, 53].

The concentration of butyrate may reach approximately 10 to 20 mM in rat and other mammalian colons [54]. Mucosal application of propionate and butyrate caused transient increases in the short-circuit current (I_SC_) at 0.5 mM and stimulated Na^+^ absorption at 50 mM in rat distal colon [58, 60]. Butyrate at 5 mM upregulated the mRNA expression of β- and γ-subunits of epithelial Na^+^ channels (ENaC) in the human colorectal cancer cell line HT-29/B6 [61]. In contrast, butyrate inhibited cAMP-mediated Cl^−^ secretion with a 50% inhibitory concentration of 8 mM [15]. Butyrate is the only SCFA to be extensively metabolized within the epithelium, leading to the production of ketone bodies, H^+^, CO_2_, and ATP. It also acts as a histone deacetylase inhibitor [18]. These factors may exert direct or indirect effects on electrolyte transport in colon.

The aim of the present study was to clarify the effects of butyrate on electrolyte transport in rat rectal colon (RC). The surface cells of the rat RC show electrogenic Na^+^ absorption through ENaC [29], which is inhibited by amiloride. We used Ussing chambers to measure amiloride-sensitive and amiloride-insensitive currents across the mucosa and demonstrated that butyrate activated K^+^ secretion through KCNQ-type K^+^ channels on the apical membrane in epithelial cells. We discussed the possibility of butyrate being transported by an electroneutral pathway via H^+^-coupled MCTs and non-ionic diffusion into rectal epithelial cells.

## Materials and methods

### Animals and tissue preparation

All experiments were performed in accordance with a protocol approved by the Laboratory Animal Care and Use Committee of The University of Tokushima and by the Animal Experimentation Committee, Kansai Medical University. Male Sprague-Dawley (SD) rats (200–425 g; *n* = 144) were fed a standard rat diet (Labo MR Standard; Na^+^ content 2.1 g/kg, K^+^ content 7.8 g/kg; Nosan Corporation, Yokohama, Japan) and tap water.

Animals were killed by cervical dislocation. Pelvic bones were immediately cut open, and a segment from distal colon (DC) to anus was excised. The rectal colon (RC) between the lymph nodes (typically located 3 cm from the anus) at the pelvic brim and anus was divided into two segments of equal lengths and the segment near the anus was used in the present study. Ileum (IL) was also excised to investigate mRNA expression. Each segment was cut open longitudinally and flushed to remove fecal pellets with standard NaCl solution containing (in mM) NaCl (145), KCl (5), CaCl_2_ (1), MgCl_2_ (1), HEPES (10; pH 7.4, adjusted with NaOH), and D-glucose (10). The epithelium (mucosa) was separated from the underlying submucosa and muscle layers by gently scraping mucosal side up along the length of the colonic segment using a glass slide [59]. We did not confirm the condition of the mucosa, but it may be similar to the “partial mucosal strip” described by Frizzell et al. [20]. When we observed the muscle layer, we turned the mucosa upside down and removed the muscle using fine forceps.

### Transepithelial current measurements

The stripped mucosa was mounted in a modified Ussing chamber with a tissue holder having an aperture surface area of 0.5 cm^2^, and then bathed bilaterally in modified Krebs-Ringer bicarbonate solution (KRB, also referred to as “124 Cl^−^” in the figure, see Table 1) consisting of (in mM) NaCl (115), KCl (5), CaCl_2_ (1), MgCl_2_ (1), NaHCO_3_ (25), HEPES (10; pH 7.4, adjusted with NaOH), and D-glucose (10) as metabolic fuel. The bathing solution was continuously gassed with a mixture of 95% O_2_–5% CO_2_ and kept at 37 °C. Unless otherwise noted, the bathing solution, which was kept at 5 ml in the chambers, was perfused at a rate of 1 ml/min with a peristaltic pump (MINIPULS 3, Gilson). Two lanes of the pump were used to input fresh solution to the apical and basolateral baths, while the other two lanes were used to pump the solution out of the baths. When drugs were applied to either side of the bathing solution, 5 μl of stock solutions were added to give the desired final concentration, immediately followed by continuous perfusion with the solution containing the appropriate concentration. Antibiotics were not needed because epithelia were continuously perfused with bathing solution. Indomethacin (5 μM) was routinely added to the solutions bathing the serosal surfaces in order to inhibit endogenous prostaglandin formation because prostaglandins are known to influence electrolyte transport across the hindgut epithelium [26].

**Table 1:** Experimental solutions in Ussing chamber experiments (all concentrations were indicated as mM)

Component	KRB (124 Cl^−^)	30 butyrate KRB	HEPES buffer	HCO_3_^−^-free butyrate
NaCl	115	85	145	115
Na-butyrate	–	30	–	30
NaHCO_3_	25	25	–	–
Component	30 formate KRB	60 butyrate KRB	10 butyrate KRB	3 butyrate KRB
NaCl	85	55	105	112
Na-butyrate	–	60	10	3
Na-formate	30	–	–	–
NaHCO_3_	25	25	25	25

All solutions contained (in mM) KCl (5), MgCl_2_ (1), CaCl_2_ (1), HEPES (10), and D-glucose (10), adjusted to pH 7.4 with NaOH (approximately 5 mM for titration), and were continuously gassed with a mixture of 95% O_2_–5% CO_2_ (for KRB or other bicarbonate buffers similar to KRB) or 100% O_2_ (for HEPES buffer and bicarbonate-free HEPES buffer)

Tissues were continuously short-circuited to monitor the short-circuit current (I_SC_, μA/cm^2^) using a voltage-clamping amplifier (CEZ-9100, Nihon Kohden Co., Tokyo) as described previously [29]. In brief, the transepithelial electrical potential difference (V_te_) was measured by a pair of pipette-shaped voltage-sensing electrodes made of a sintered Ag-AgCl pellet filled with a solution of 3% (*w*/*v*) agarose in 3 M KCl solution. A transepithelial current was passed across the tissue through a pair of pipette-shaped electrodes made of Ag wire (Physiologic Instruments) filled with a solution of 3% (*w*/*v*) agarose in 3 M KCl solution. I_SC_ was referred to as positive for cations flowing across the epithelium from the mucosal side to the serosal side. All preparations were allowed to equilibrate for 40–90 min after mounting on the chambers before measurements were taken. Transepithelial resistance (R_t_) was typically estimated from the current change in response to square voltage pulses (+ 2 mV, 1-s duration) imposed across the mucosa at 2-min intervals or manually. The resistance of the bathing fluid between the voltage-sensing electrodes was measured and compensated for by the amplifier before each experiment. In order to avoid using tissues that were damaged during preparation, we only proceeded with experiments when initial R_t_ values were larger than 50 Ω cm^2^. The averaged R_t_ values in the present study, approximately 90 Ω cm^2^, i.e., 11.1 mS/cm^2^ (see Results), were similar to those previously reported for the rat colon [5, 28–30, 35]. The potential difference in the voltage electrodes was less than ± 1.0 mV during the period of measurements. Stock solutions of drugs were applied to give the desired final concentration. In order to replace solution, 2.5 ml of solution was withdrawn from the apical and basolateral bathing solutions and each was added successively with an equal volume. This procedure was repeated 10 times in parallel for continuous perfusion with the bathing solution. I_SC_, amiloride-sensitive I_SC_ (I_SC-amil/sen_), and R_t_ were stable during the experiments [30]. We defined amiloride-insensitive I_SC_ as I_SC-amil/insen_. In order to estimate the effects of butyrate on I_SC-amil/insen_, we subtracted I_SC-amil/insen_ in KRB from that in 30 butyrate KRB and defined the yield as the I_SC-amil/insen_ shift. We applied bumetanide, a Na^+^-K^+^-2Cl^−^ cotransporter inhibitor, to the basolateral bath and verified the accuracy of the recording at the end of experiments. We ceased perfusion in this protocol to save the chemical.

### Curve fitting

The dependency of the amiloride-insensitive short-circuit current (I_SC-amil/insen_) shift on substitute (butyrate or XE991) concentrations was fit using the following Hill equation:

$$ \mathrm{I}={\mathrm{I}}_0+\left({\mathrm{I}}_{\mathrm{max}}-{\mathrm{I}}_0\right)/\left\{1+{\left(\mathrm{S}/{\mathrm{K}}_{\mathrm{D}}\right)}^n\right\}, $$

where I, I_0_, and I_max_ are the I_SC-amil/insen_ shift as a function of the substitute concentration, the residual I_SC-amil/insen_ shift independent of the substitute concentration, and the I_SC-amil/insen_ shift (extrapolated) at the maximal substitute concentration, respectively. S is the substitute concentration, K_D_ is the dissociation constant (for XE991, K_i_ is used instead as the inhibitory constant), that is, the substitute concentration of the half-maximal I_SC-amil/insen_ shift, and *n* is the Hill coefficient.

### Chemicals

All chemicals employed were reagent grade. Amiloride and bumetanide were obtained from Sigma (St. Louis, MO). Sodium butyrate and sodium formate were obtained from Wako Chemicals (Osaka, Japan). HEPES, indomethacin, and ibuprofen were obtained from Nacalai Tesque (Kyoto, Japan). XE991 (10, 10-bis (4-pyridinylmethyl)-9(10H)-anthracenonedihydrochloride) was obtained from Alomone Labs (Israel). Chromanol 293B and bupivacaine were obtained from Tocris Bioscience (Bristol, UK) and Tokyo Chemical Industry (Tokyo, Japan), respectively. Stock solutions of amiloride (10 mM), XE991 (100 mM), and barium chloride (1 M) were prepared in distilled water. Indomethacin (5 mM) and ibuprofen (300 mM) were dissolved in ethanol. Bumetanide (100 mM), chromanol 293B (100 mM), and bupivacaine (300 mM) were dissolved in dimethyl sulfoxide (DMSO). Stock solutions were prepared at a 1000-fold concentration for administration, except for barium chloride at a 200-fold concentration.

### Statistical analysis

Results were reported as the means ± SE of several experiments (*n*). The significance of differences was evaluated using the two-tailed paired and unpaired Student’s *t* test. Differences between means were considered to be significant at a value of *p* < 0.05.

### RT-PCR analysis

RNA was extracted from the mucosae of rat rectal colon (RC), distal colon (DC), and ileum (IL) using TRIzol reagent (Invitrogen, Carlsbad, CA, USA), treated with RQ1 RNase-free DNase (Promega, Madison, WI, USA), extracted with phenol–chloroform, precipitated with ethanol, and then resuspended in ultra-pure water. First-strand cDNA was generated using SuperScript III RT (Invitrogen). PCR analyses were performed using Phusion High-Fidelity DNA Polymerase (Finnzymes, Vantaa, Finland) with primers designed to recognize different types of transporters and channels (Table 2). The amplification parameters used were as follows: one cycle at 98 °C for 30 s, followed by 35 cycles at 98 °C for 5 s, 63–64 °C for 10 s, 72 °C for 15 s, and one cycle at 72 °C for 5 min. The transcripts were subsequently checked by agarose gel electrophoresis.

**Table 2:** Primer sets used for the rat intestine in the RT-PCR analysis

Gene (subunit)		Size (bp)
Slc16a1 (MCT1)		
Forward:	5′-TGGTATTTTTGGCTGGAGAGG-3′	859
Reverse:	5′-TTTACCGTCCCTCTTCTTTTC-3′	
Slc16a3 (MCT4)		
Forward:	5′-GGCAGTCCCGTGTTCCTTTG-3′	929
Reverse:	5′-ACCACCTCCCCGTTTTTCTC-3′	
Slc16a4 (MCT5)		
Forward:	5′-ACGATTGGGTCTTTCTACAGC-3′	880
Reverse:	5′-GGCATATCCCAAAAACCTGTG-3′	
Slc16a8 (MCT3)		
Forward:	5′-ATGCTGGCTATGCTCTACGG-3′	717
Reverse:	5′-GGCCAGGCTGAAGAGATAGG-3′	
Kcnq1 (Kv7.1)		
Forward:	5′-AGGTGCTATGCTGCGGAGAA-3′	613
Reverse:	5′-CACGGTCTTTGCTCTTTTCTGA-3′	
Kcnq2 (Kv7.2)		
Forward:	5′-TACGGGCCAGAGGAAATACG-3′	510
Reverse:	5′-GCCCAAGCCACATCTCCAAAG-3′	
Kcnq3 (Kv7.3)		
Forward:	5′-GGTTCGCCTTTCTAATCCTCGT-3′	790
Reverse:	5′-GGGGCCTGATTCTGAGTAGTT-3′	
Kcnq4 (Kv7.4)		
Forward:	5′-GATGATCGTGGTCTTTGGTTTG-3′	664
Reverse:	5′-CCGGCTCGTGTCAGTGGAAT-3′	
Kcnq5 (Kv7.5)		
Forward:	5′-AGCCATCAAGCATCTATCCAGG-3′	502
Reverse:	5′-ATCCGTACTGTCTCCCGTCTTA-3′	
Kcne1		
Forward:	5′-GGCGGACTTGGCTCGTAGG-3′	281
Reverse:	5′-GTGTGGCAGGTTGTTCTACG-3′	
Kcne2		
Forward:	5′-GACAGCTGGAGGAGGAACACA-3′	224
Reverse:	5′-CTCCTATACTTCTGCTGCCAATC-3′	
Kcne3		
Forward:	5′-TGCTAAAGGCTCTGAACACAAC-3′	232
Reverse:	5′-TCACTACGTTTGTCCACTTTGC-3′	
Kcne4		
Forward:	5′-ATGGAGCCTCTGAATAGCACAC-3′	476
Reverse:	5′-GACCCTTCGCTGCTTTCATTG-3′	
Kcne5		
Forward:	5′-AACCCTCTTGAACCGCTTGC-3′	228
Reverse:	5′-TTGCGGGAGCGAGTGTAGG-3′	

### Immunolocalization

Immunolocalization was performed on the rat rectal colon. The rectal colon was obtained from male Sprague-Dawley and Wistar rats (*n* = 3). Protocols involving the handling of animals were approved by the Animal Experimentation Committee, Kansai Medical University. Rats were anesthetized with isoflurane and a mixture of medetomidine (0.4 mg/kg body weight), midazolam (2.0 mg/kg b.w.), and butorphanol (5.0 mg/kg b.w.), and perfused transcardially with 4% paraformaldehyde in phosphate-buffered saline (PBS). The rectal colon was removed, cut into small pieces, fixed with 4% paraformaldehyde in PBS for 24 h, embedded in paraffin, and sectioned. Detailed methods for immunohistochemistry are described elsewhere [27]. Briefly, the specimen was permeabilized with 0.2% Triton X-100 in PBS for 10 min. Autofluorescence was blocked in 0.1 M Tris-glycine. Non-specific binding was blocked with 2% normal donkey serum in PBS and incubated with primary antibodies (1:100 to 1:400) with antibodies for Ezrin (1:400, clone 3C12, MS-661; Lab Vision) in immunoreaction enhancer solution (Can Get Signal immunostain; Toyobo) at 4 °C overnight. Secondary antibodies conjugated to Alexa488 or Alexa568 (1:400; Molecular Probes) were added for 1 h. Antibodies for KCNQ1 (APC-022), KCNQ2 (APC-050), KCNQ4 (APC-164), and KCNE3 (APC-118) were obtained from Alomone Labs (Israel). As the negative control, primary antibodies were preincubated with control peptide antigens (Alomone Labs) at room temperature for 30 min and scanning was performed using the same settings. Nuclei were stained with 4′, 6-diamidino-2-phenylindole (DAPI) at 1 μg/ml. Fluorescence was observed with a confocal laser scanning microscope (LSM510 META; Carl Zeiss).

## Results

### Butyrate induced an amiloride-insensitive short-circuit current in rat rectal colon

**Fig. 1:**
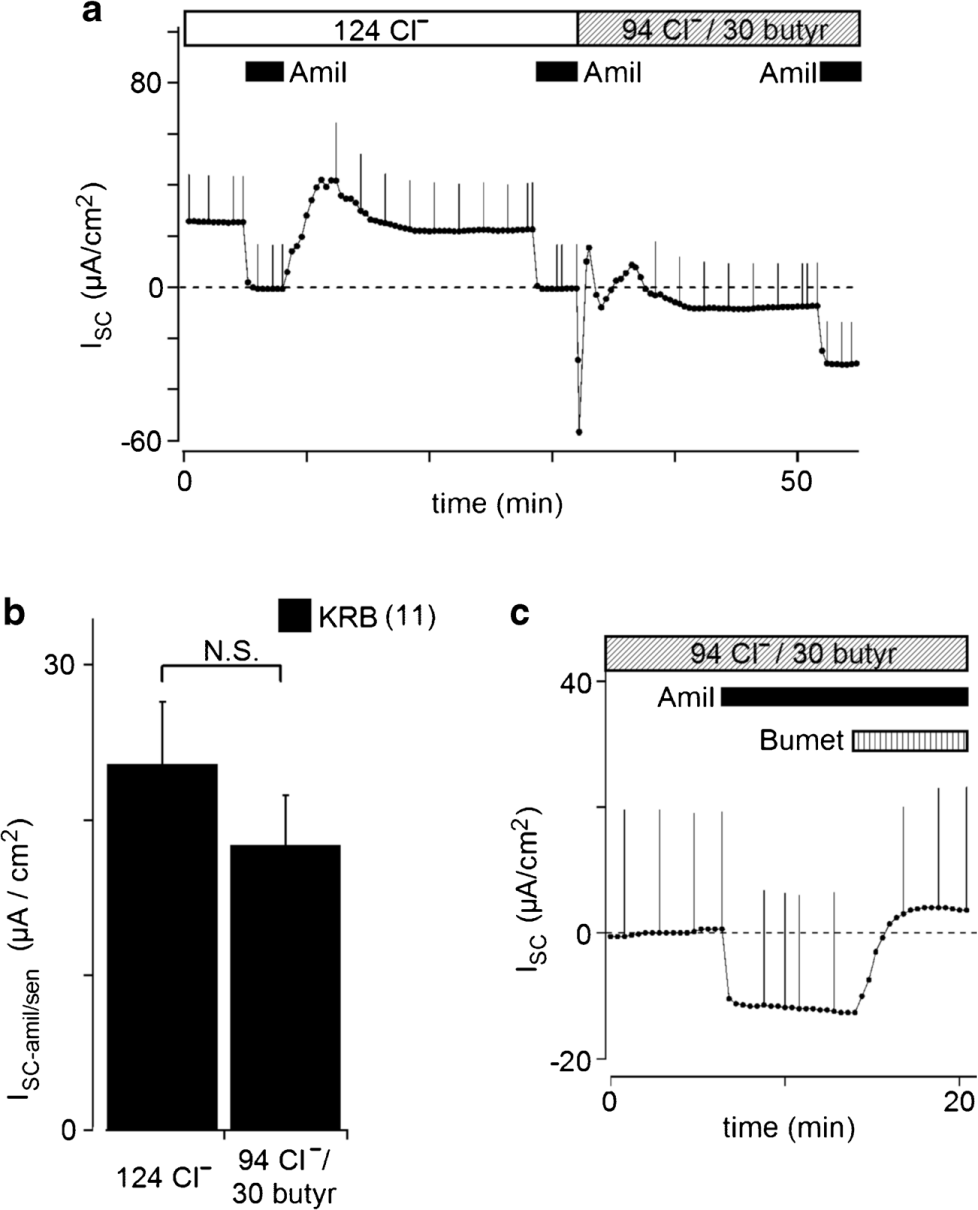
Effects of butyrate on I_SC_. **a** Effects of butyrate applied to the apical and basolateral sides on short-circuit currents (I_SC_) in rat rectal colonic mucosa. Current deflections are caused by a 2-mV voltage pulse injection every 2 min or by manual insertion. Bars on the top of this figure indicate the perfusion solution. Filled boxes indicate the timing of the apical application of amiloride (Amil; 10 μM). A positive deflection in I_SC_ indicates cation movement from the apical to basolateral bathing solution or anion movement from the basolateral to apical solution. 124 Cl^−^ indicates KRB and 94 Cl^−^/30 butyrate indicates 30 butyrate KRB (see Table 1). **b** Effects of butyrate on amiloride-sensitive I_SC_ (I_SC-amil/sen_). No significant differences were observed in I_SC-amil/sen_ between control and 30 mM butyrate KRB. Values are means ± SE (*n* = 11). **c** Representative experiment of the basolateral administration of 100 μM bumetanide after the application of amiloride with butyrate. Major anion compositions are shown on top of the trace (see Table 1). The shaded box indicates the basolateral application of bumetanide (Bumet; 100 μM). The trace only shows after the third amiloride application in 30 butyrate-KRB, as shown in Fig. 1a, for clarity

Surface cells of rat rectal colon express ENaC, which is responsible for electrogenic Na^+^ absorption [29]. Thus, we used amiloride, a blocker of ENaC, to assess whether butyrate affected the activity of ENaC measured as an amiloride-sensitive short-circuit current (I_SC-amil/sen_) in the Ussing chamber. Figure 1a shows an example of control experiments. When amiloride (10 μM) was added to the apical solution, I_SC-amil/sen_ was 23.1 ± 1.7 μA/cm^2^ (*n* = 73) and the values obtained for transepithelial resistance (R_t_) significantly increased from 89.7 ± 2.0 to 91.5 ± 2.0 Ω cm^2^ (*p* < 0.0001; *n* = 73). During two consecutive applications of amiloride, the first and second I_SC-amil/sen_ were 24.1 ± 4.2 and 20.7 ± 3.7 μA/cm^2^, respectively (*n* = 7). These stable and reproducible responses to amiloride ensured similar basal I_SC-amil/sen_; therefore, we examined the effects of butyrate on the second I_SC-amil/sen_ in different mucosal preparations, as described in previous studies [29, 30].

When 30 mM butyrate (30 butyrate KRB, Table 1) was applied to both sides of the mucosa (Fig. 1a), I_SC_ showed transient negative, followed by positive changes, as reported previously [37], and then a negative shift. This negative shift indicated a decrease in cation absorption or anion secretion and/or an increase in cation secretion or anion absorption. In order to assess cation absorption through ENaC, we added 10 μM amiloride to the apical bath. I_SC-amil/sen_ in 30 butyrate KRB, 18.4 ± 3.2 μA/cm^2^, was not significantly different from that in KRB (*n* = 11, Fig. 1b), suggesting that butyrate did not attenuate the activity of ENaC. We measured I_SC_ with 10 μM amiloride (I_SC-amil/insen_) in KRB and 30 butyrate KRB. The subtraction of I_SC-amil/insen_ in KRB from I_SC-amil/insen_ in 30 butyrate KRB yielded an I_SC-amil/insen_ shift induced by 30 mM butyrate of −29.5 ± 2.7 μA/cm^2^ (*n* = 11). In preliminary experiments, we found that acetate caused a similar I_SC-amil/insen_ shift.

Na^+^-K^+^-2Cl^−^ cotransporters on the basolateral membrane uptake Na^+^, K^+^, and Cl^−^ into the surface cells of the colonic epithelium and provide sufficient intracellular Cl^−^ and K^+^ for secretion [48, 50]. The application of bumetanide (100 μM), an inhibitor of Na^+^-K^+^-2Cl^−^ cotransporter, to the basolateral side in addition to butyrate and apical amiloride returned I_SC-amil/insen_ to nearly zero, +0.3 ± 2.6 μA/cm^2^ (*n* = 5; Fig. 1c). Thus, the butyrate-dependent I_SC-amil/insen_ shift may result from an increase in K^+^ uptake across basolateral membrane and K^+^ efflux across apical membrane.

### Effects of K^+^ channel inhibitors on the I_SC-amil/insen_ shift

**Fig. 2:**
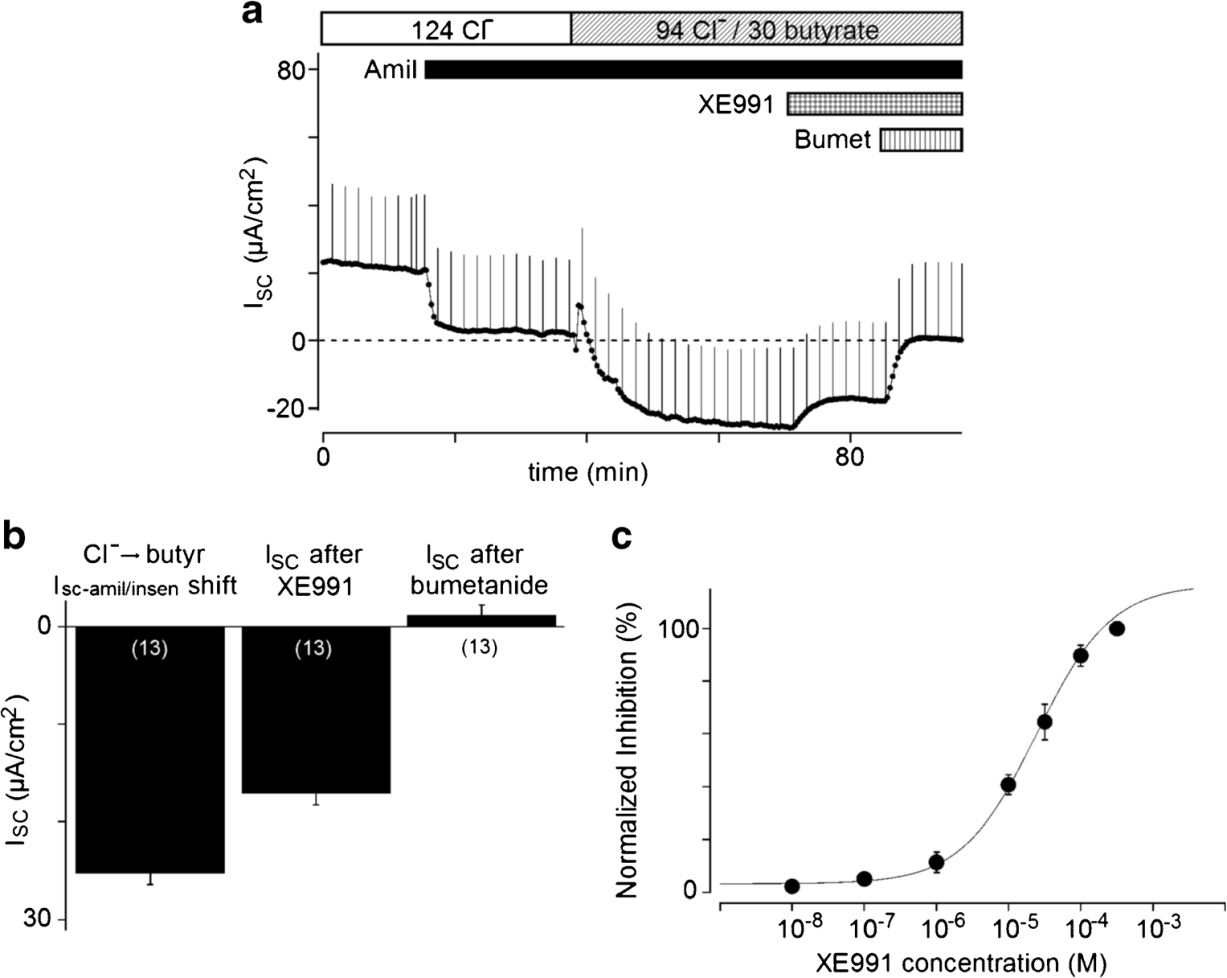
Effects of XE991 on I_SC_. **a** Representative experiment of the apical administration of 100 μM XE991 after the application of amiloride and butyrate. **b** A summary of the effects of XE991 on I_SC_. Bars refer to the cumulative addition of drugs shown in Fig. 2a. **c** Dose dependency of XE991 for the shift in I_SC-amil/insen_ (*n* = 5). The solid curve shows the fit of results using the Hill equation (see the "Materials and methods" section). Values are the means ± SE of five experiments

In order to identify the K^+^ secretion pathway on the apical membrane, we tested the effects of K^+^ channel inhibitors on the I_SC-amil/insen_ shift by butyrate. We maintained the concentration of amiloride at 10 μM in the bathing solution. The apical application of 100 μM XE991, a KCNQ-type K^+^ channel inhibitor, significantly decreased the I_SC-amil/insen_ shift (−8.2 ± 0.9 μA/cm^2^, *n* = 13; Fig. 2a). The value of the I_SC-amil/insen_ shift induced by butyrate was −25.3 ± 1.1 μA/cm^2^. XE991 (100 μM) increased I_SC__-amil/insen_ to −17.1 ± 1.2 μA/cm^2^, and bumetanide (100 μM) further increased I_SC-amil/insen_ to 1.2 ± 1.0 μA/cm^2^ (*n* = 13; Fig. 2a, b). XE991 inhibited the I_SC-amil/insen_ shift in a concentration-dependent manner and the relationship was fit well by the Hill equation (Fig. 2c), which estimated K_i_ and *n* as 34 ± 19 μM and 0.87 ± 0.07 (*n* = 5), respectively. In control KRB solution, 100 μM XE991 slightly decreased I_SC_ by −1.6 ± 0.8 μA/cm^2^ (*n* = 5). In preliminary experiments, I_SC__-amil/insen_ in 30 butyrate KRB was not affected by chromanol 293B (KCNQ1 inhibitor, 100 μM, *n* = 2), paxilline (BK channel inhibitor, 100 nM, *n* = 2), TRAM34 (K_Ca_ 3.1 inhibitor, 1 μM, *n* = 2), or bupivacaine (two-pore domain K^+^ channel inhibitor, 300 μM, *n* = 3) (not shown). Additionally, the apical application of 5 mM Ba^2+^, a non-specific K^+^ channel inhibitor, did not inhibit the I_SC-amil/insen_ shift (*n* = 5; not shown).

### Effects of formate on the I_SC-amil/insen_ shift

**Fig. 3:**
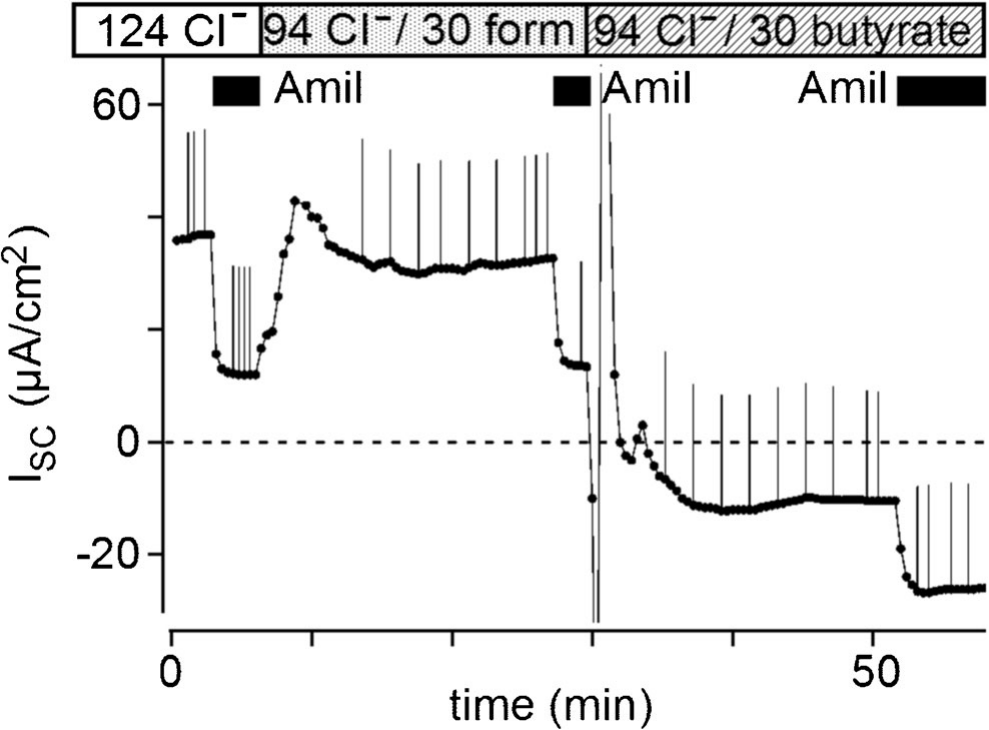
Effects of formate (methanoate) on the short-circuit current (I_SC_) in rat rectal colon. In order to clarify the shift in amiloride-insensitive I_SC_, a part of the transient direction was deleted from the trace

In the experiments described above, the chloride concentration was reduced from 124 to 94 mM in 30 butyrate KRB. In order to examine whether reductions in chloride concentrations affect the butyrate-induced I_SC-amil/insen_ shift, we measured I_SC_ in a solution containing 30 mM formate (methanoate) (Fig. 3). In 30 formate KRB, I_SC-amil/sen_ was 15.0 ± 2.7 μA/cm^2^ and I_SC-amil/insen_ was 5.3 ± 3.7 μA/cm^2^ (*n* = 5). I_SC-amil/insen_ in 30 formate KRB was not significantly different from that in KRB (5.5 ± 1.8 μA/cm^2^, *n* = 7). The substitution to 30 butyrate KRB from 30 formate KRB caused an I_SC-amil/insen_ shift of −29.1 ± 3.7 μA/cm^2^ (*n* = 5), which was similar to that in the control experiments shown in Fig. 1. These results suggest that the I_SC-amil/insen_ shift induced by butyrate was not due to a reduction in the chloride concentration.

### Dependency of the I_SC-amil/insen_ shift on butyrate concentrations

**Fig. 4:**
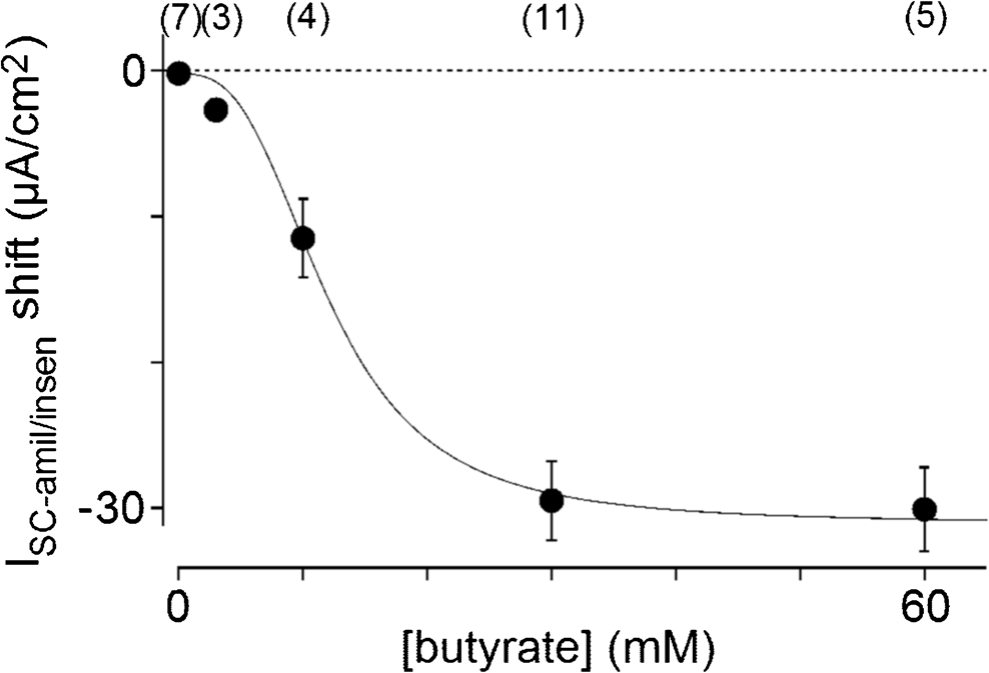
Concentration dependency of butyrate for the shift in amiloride-insensitive I_SC_ (I_SC-amil/insen_ shift). The solid curve shows the fit of results using the Hill equation (see the "Materials and methods" section)

We examined the dependency of the I_SC-amil/insen_ shift on butyrate concentrations. When 0 (only performed with the solution exchange with KRB), 3, 10, 30, and 60 mM butyrate were applied on both sides after the first application of amiloride, the I_SC-amil/insen_ shifts observed were −0.2 ± 0.3 (*n* = 7), −2.7 ± 0.5 (*n* = 3), −11.5 ± 2.7 (*n* = 4), −29.5 ± 2.7 (*n* = 11), and −30.1 ± 2.9 (*n* = 5) μA/cm^2^, respectively (Fig. 4). The I_SC-amil/insen_ shift showed dependency on the butyrate concentration and the relationship was fit well by the Hill equation, which estimated K_D_ and *n* as 12.0 mM and 2.9, respectively.

### Transport pathway for butyrate into colonic epithelial cells

**Fig. 5:**
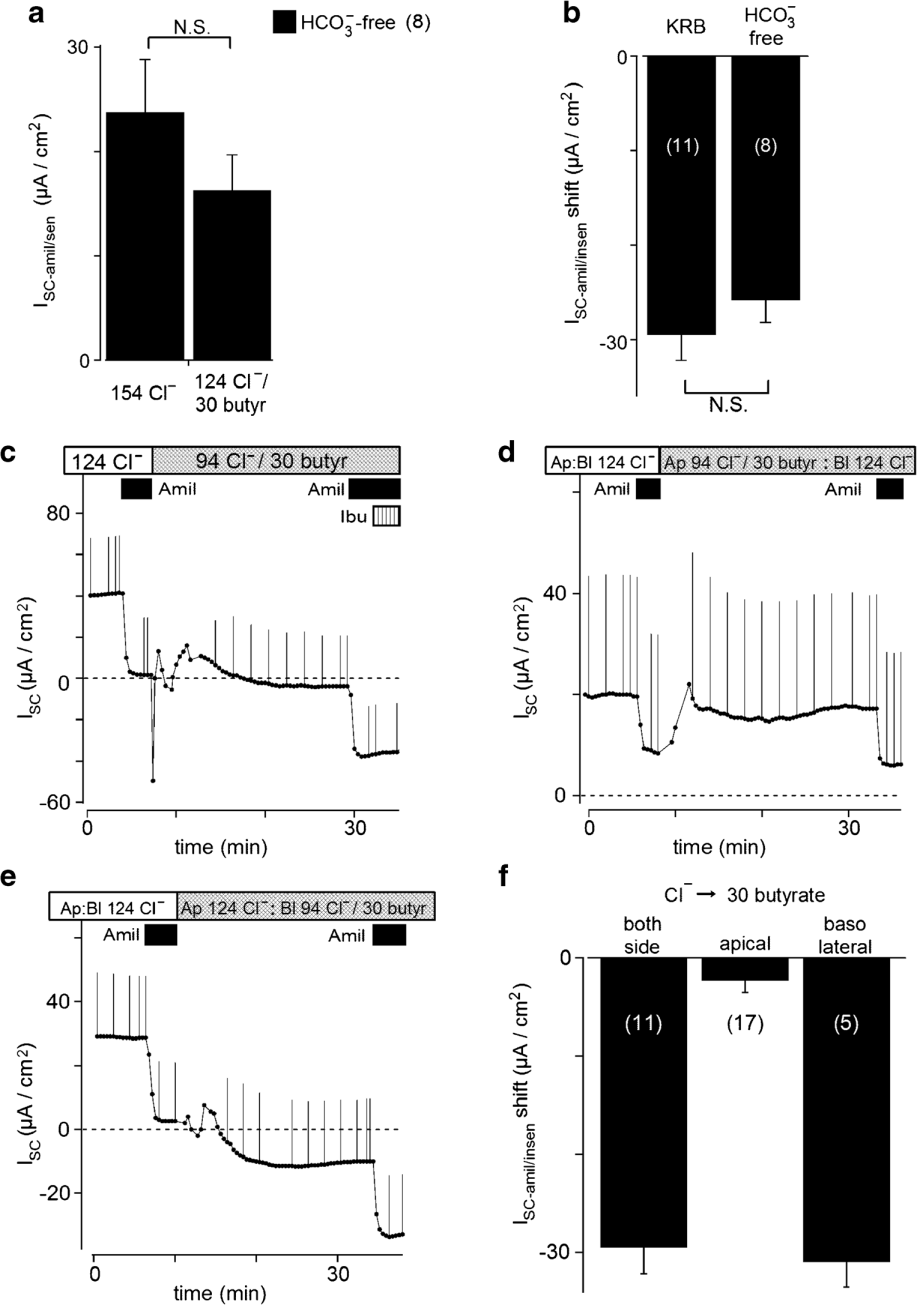
**a** Effects of butyrate on amiloride-sensitive I_SC_ (I_SC__-amil/sen_) without HCO_3_^−^ (substituted for NaCl with O_2_ bubbling). No significant differences were observed in I_SC__-amil/sen_ between control and 30 mM butyrate without HCO_3_^−^. Under HCO_3_^−^-free conditions, the epithelium did not have the endurance required for a long-term experiment with the third amiloride application; therefore, this comparison was performed on the second amiloride application. Values are the means ± SE (*n* = 8). **b** Effects of butyrate on the shift in amiloride-insensitive I_SC_ (I_SC-amil/insen_ shift). No significant differences were observed in I_SC-amil/insen_ shift with or without HCO_3_^−^ (substituted for NaCl with O_2_ bubbling). **c** Ibuprofen (Ibu; 300 μM) did not affect I_SC_ after the application of amiloride with butyrate. **d** Effects of 30 mM butyrate applied to the apical side on short-circuit currents (I_SC_) in rat rectal colonic mucosa. **e** Effects of 30 mM butyrate applied to the basolateral side on I_SC_. **f** Averaged I_SC-amil/insen_ shift by the application of butyrate to the apical, basolateral, or both sides. Values are the means ± SE. Numbers in parentheses refer to the number of experiments

SCFA^−^/HCO_3_^−^ exchangers and Na^+^-coupled transporters for monocarboxylates (SMCT1) are candidates for the electrogenic SCFA transport pathway on colonic epithelia. In HCO_3_^−^-free solution, I_SC-amil/sen_ were 23.8 ± 5.0 and 16.3 ± 3.4 μA/cm^2^ with 0 and 30 mM butyrate, respectively (Fig. 5a). The I_SC-amil/insen_ shift was also observed in HCO_3_^−^-free solution with a value of −25.8 ± 2.4 μA/cm^2^ (*n* = 8), which was not significantly different from that obtained under HCO_3_^−^ conditions in Fig. 1 (−29.5 ± 2.7 μA/cm^2^, *n* = 11; Fig. 5b). Ibuprofen at 300 μM, which inhibited a propionate-evoked current through SMCT1 with a K_i_ value of 70 μM [42], had no effects on I_SC-amil/insen_ in 30 butyrate KRB (*n* = 6; Fig. 5c). Therefore, it is unlikely that SCFA^−^/HCO_3_^−^ exchangers and SMCT1 were involved in the I_SC-amil/insen_ shift induced by butyrate.

The apical application of 30 mM butyrate only induced a markedly reduced I_SC-amil/insen_ shift (−2.3 ± 1.2 μA/cm^2^, *n* = 17; Fig. 5d, f). These values may have been underestimated because we did not compensate for liquid junction potential in the transepithelial electrical potential difference. In contrast, the basolateral application of 30 mM butyrate caused an I_SC-amil/insen_ shift with a value of −31.0 ± 2.5 μA/cm^2^ (*n* = 5; Fig. 5e, f).

### Expression of MCTs in rat rectal colon

**Fig. 6:**
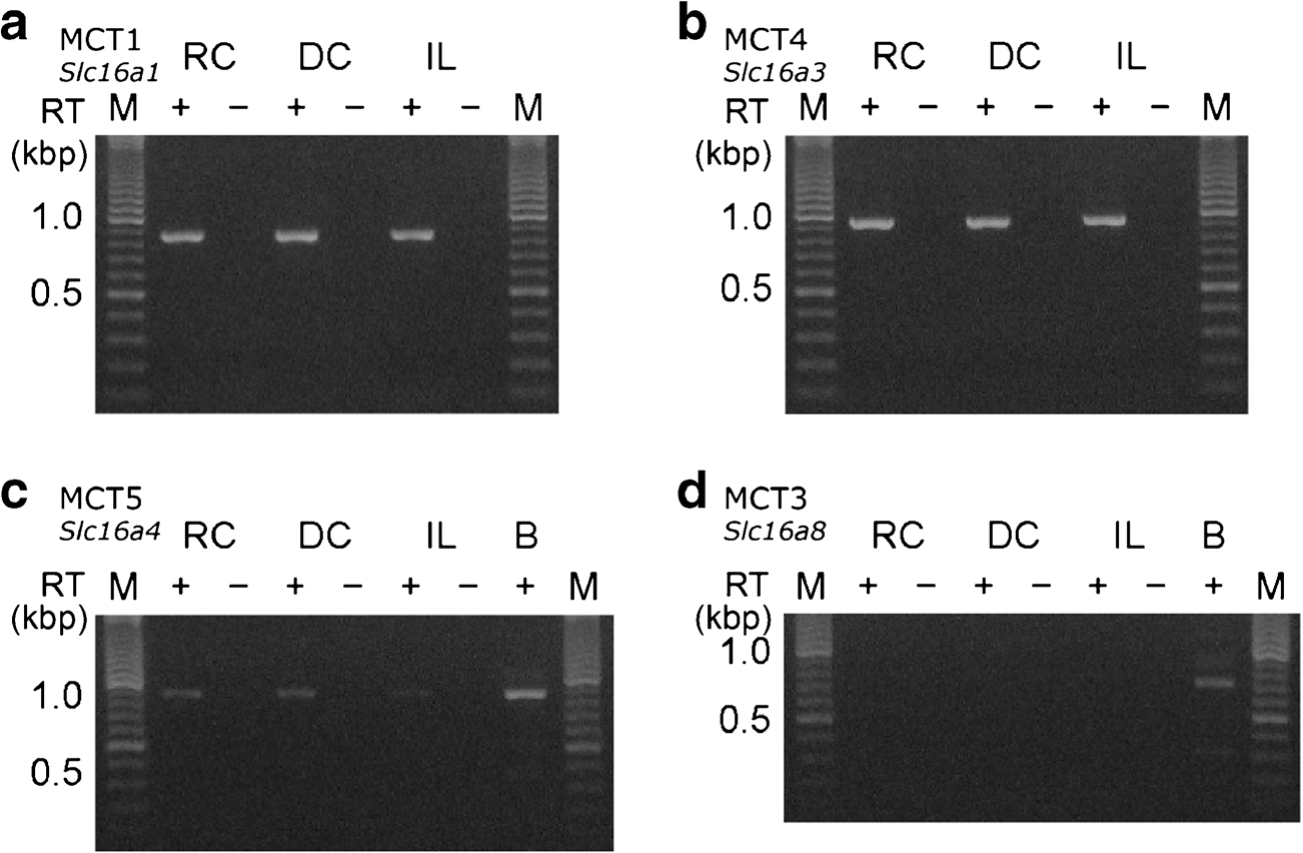
RT-PCR analysis of H^+^-coupled monocarboxylate transporters. An ethidium bromide-stained agarose gel shows RT-PCR products generated from total RNA isolated from the mucosa of rectal colon (RC), distal colon (DC), and ileum (IL). The primers used gave the expected fragment lengths for *Slc16a1* (859 bp; **a**), *Slc16a3* (929 bp; **b**), *Slc16a4* (880 bp; **c**), and *Slc16a8* (717 bp; **d**). First-strand cDNA was generated with SuperScript III reverse transcriptase (RT, +). No DNA fragment was amplified with the template without reverse transcription (−). B in panels **c** and **d**, positive controls obtained from brain. A representative gel for at least three independent experiments is shown. M, molecular mass

The expression of MCTs on the isolated rat mucosa was confirmed using an RT-PCR analysis. Figure 6 shows that the mucosae of the rectal colon (RC), distal colon (DC), and ileum (IL) expressed *Slc16a1* (MCT1), *Slc16a3* (MCT4), and *Slc16a4* (MCT5), but not *Slc16a8* (MCT3) (*n* = 3 animals).

### Expression of α- and β-subunits of KCNQ channels in rat rectal colon

**Fig. 7:**
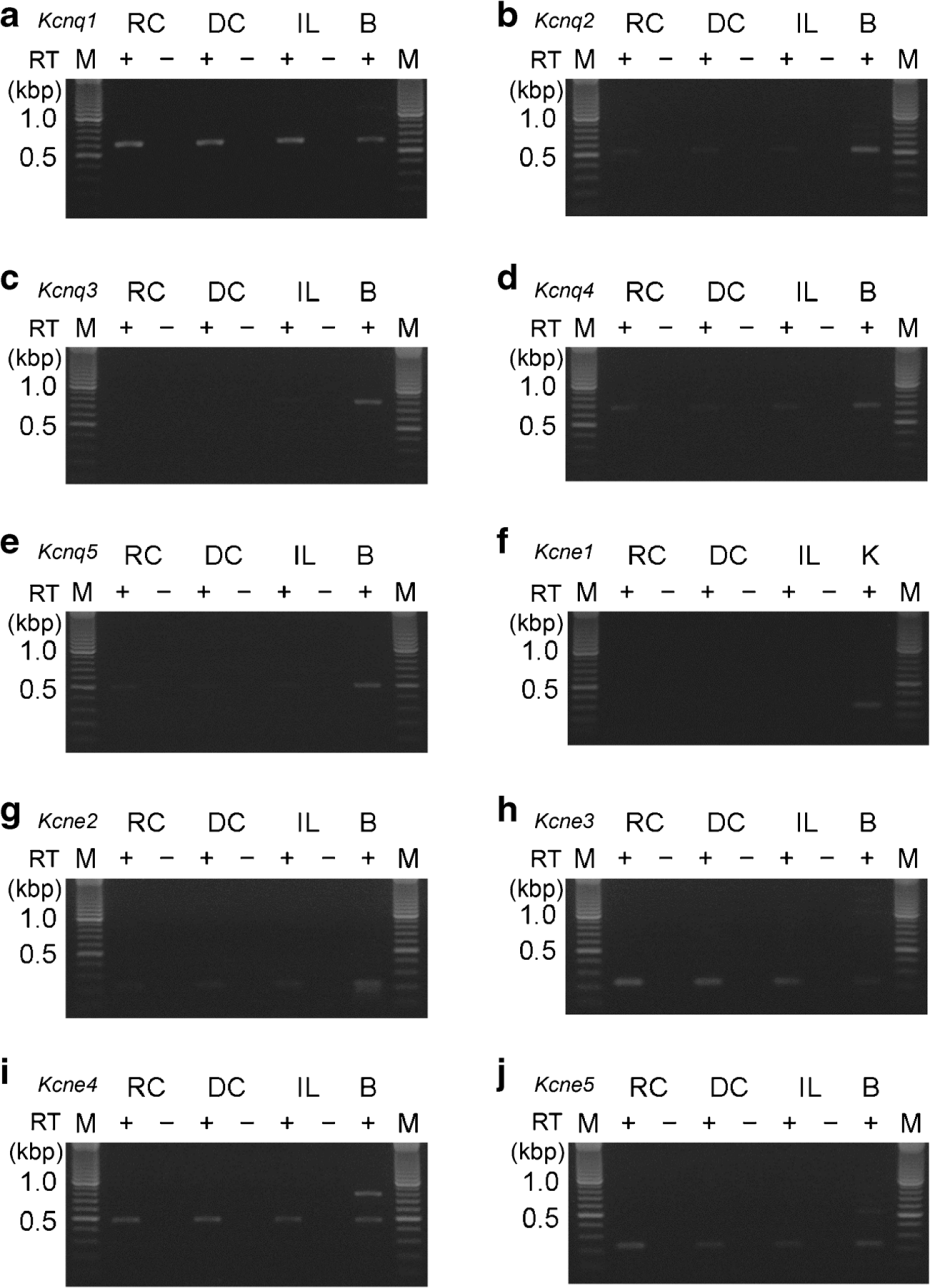
RT-PCR analysis of α- and β-subunits of KCNQ channels from the mucosa of rectal colon (RC), distal colon (DC), and ileum (IL). The primers used gave the expected fragment lengths for *Kcnq1* (613 bp; **a**), *Kcnq2* (510 bp; **b**), *Kcnq3* (790 bp; **c**), *Kcnq4* (664 bp; **d**), *Kcnq5* (502 bp; **e**), *Kcne1* (281 bp; **f**), *Kcne2* (224 bp; **g**), *Kcne3* (232 bp; **h**), *Kcne4* (476 bp; **i**), and *Kcne5* (228 bp; **j**). RNA was treated with (+) or without (−) reverse transcriptase (RT). Positive controls obtained from brain (B) or kidney (K). A representative gel for at least three independent experiments is shown. M, molecular mass

The apical addition of XE991, a KCNQ-type K^+^ channel inhibitor, reduced the I_SC-amil/insen_ shift. Thus, we examined the expression of the α- and β-subunits of KCNQ channels on the isolated rat mucosa using a RT-PCR analysis. Figure 7 shows that the mucosae of rectal colon (RC), distal colon (DC), and ileum (IL) expressed *Kcnq1* (K_v_7.1), *Kcnq2* (K_v_7.2), *Kcnq4* (K_v_7.4), *Kcnq5* (K_v_7.5), *Kcne2*, *Kcne3*, *Kcne4*, and *Kcne5*; however, *Kcnq3* (K_v_7.3) and *Kcne1* were not detected (*n* = 3 animals).

### Immunolocalization of KCNQ subunits in rat rectal colon

**Fig. 8:**
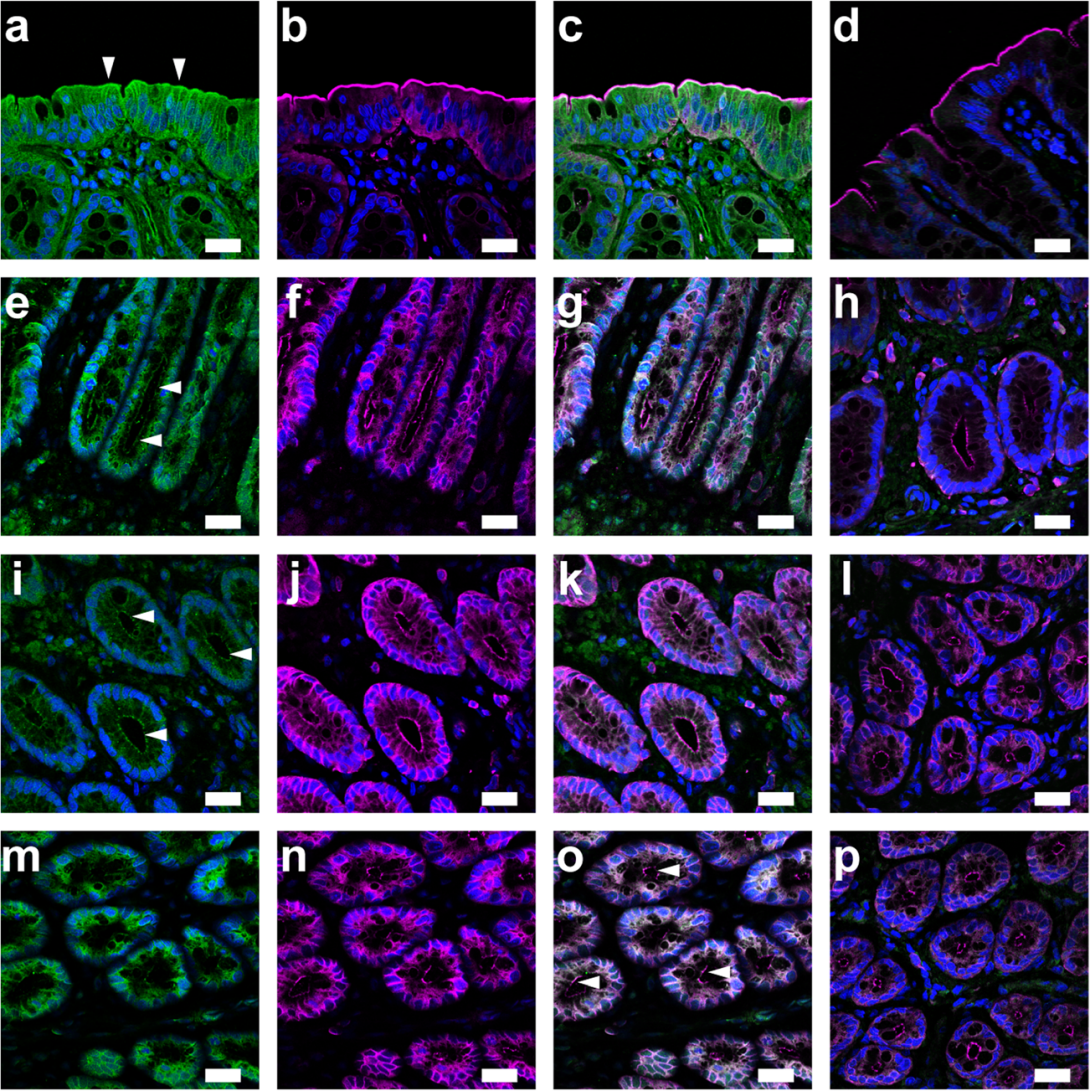
Immunolocalization of α- and β-subunits of KCNQ channels in the rat rectal colon. **a** Fluorescence of KCNQ2 on the luminal membranes of surface cells (*arrowheads*). **b** Fluorescence image of ezrin. **c** Overlay image of **a** and **b**. **d** Overlay image of ezrin and green fluorescence with KCNQ2 antibody pre-absorbed with the control peptide antigen. **e** Fluorescence of KCNQ4 on the luminal membrane of crypt cells (*arrowheads*). Fluorescence images of ezrin (**f**), the overlay (**g**), and negative control with the pre-absorbed KCNQ4 antibody (**h**). **i** Fluorescence of KCNE3 on the luminal membrane of crypt cells (*arrowheads*). Fluorescence images of ezrin (**j**), the overlay (**k**), and negative control with the pre-absorbed KCNE3 antibody (**l**). Fluorescence of KCNQ1 (**m**), ezrin (**n**), and the overlay (**o**) in crypts. *Arrowheads* show the luminal membrane of crypt cells. **p** Negative control with the pre-absorbed KCNQ1 antibody. DAPI was used to stain nuclei (blue). Bars = 20 μm

The immunolocalization of KCNQ subunits was examined with paraffin sections of the rat rectal colon (*n* = 3 animals). Immunofluorescence ascribed to KCNQ2 colocalized with Ezrin, an A-kinase anchoring protein, to the luminal membrane of surface cells in rat rectal colon (Fig. 8a–c). Immunofluorescence on the luminal membranes was diminished with KCNQ2 antibody, which was pre-absorbed with the control peptide antigen for the negative control (Fig. 8d). Additionally, immunofluorescence in the luminal membrane of surface cells was detected by antibodies against KCNQ4 (not shown). Antibodies against the KCNQ4 and KCNE3 subunits showed immunofluorescence in the luminal membranes of crypt cells (Fig. 8e–g and i–k, respectively). Immunofluorescence in the crypt cells was also detected by antibodies against KCNQ2 (not shown). However, antibodies against the KCNQ1 subunit did not show immunofluorescence in the luminal membrane, but did in the basolateral membrane of crypt cells (Fig. 8m–o), as reported previously [48]. Immunofluorescence was reduced when antibodies were pre-absorbed with the control peptide antigens (Fig. 8l, p).

## Discussion

### Possible involvement of KCNQ-type K^+^ channels in cation secretion by butyrate in rat rectal colon

**Fig. 9:**
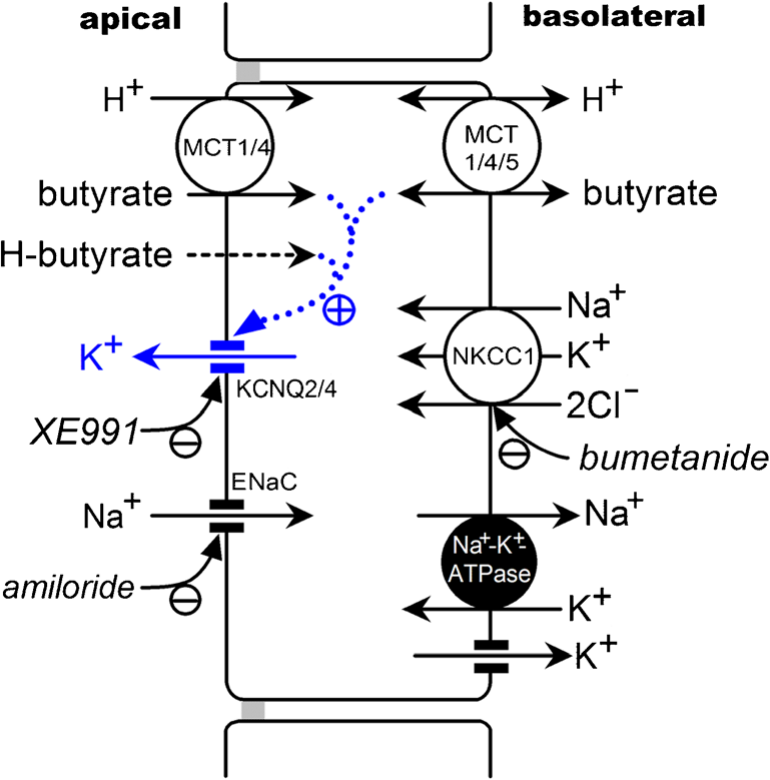
Proposed model for the regulation of K^+^ secretion by butyrate in rat rectal colon. Butyrate was transported into epithelial cells via monocarboxylate transporter 1 and/or 4 (MCT1/4) and non-ionic diffusion via lipid membrane and affected K^+^ secretion through KCNQ-type K^+^ channels on the apical membrane

In the present study, we demonstrated that butyrate regulated K^+^ secretion through KCNQ-type K^+^ channels in the apical membrane of rat rectal colon (Fig. 9). This conclusion was based on the following main results: the application of butyrate elicited a shift in I_SC-amil/insen_ in a negative direction and in a concentration-dependent manner; the butyrate response was inhibited by a KCNQ-type K^+^ channel inhibitor and Na^+^-K^+^-2Cl^−^ cotransporter inhibitor; a RT-PCR analysis confirmed the expression of KCNQ and KCNE subunits; an immunohistochemical analysis showed immunoreactivity for KCNQ2 and KCNQ4 in the luminal membranes of surface and crypt cells.

The apical addition of XE991, a KCNQ-type K^+^ channel inhibitor, inhibited the I_SC-amil/insen_ shift with a K_i_ value of 34 μM. The K^+^ channel proteins of KCNQ2 and KCNQ4 were detected in the luminal membranes of surface and crypt cells in rat rectal colon, whereas the KCNQ1 protein was localized to the basolateral membrane of crypt cells (Fig. 8). Immunoreactivity for KCNQ4 was observed in muscle layers in murine distal colon as well as in crypt cells [33]. In addition, XE991 increased the integral of tension in a segment of distal colon circular muscle exhibiting spontaneous phasic contractions. Further studies will be required in order to investigate the combination of these subunits and elucidate the physiological role of KCNQ channels in colon.

Although XE991 inhibited 32% of butyrate-stimulated K^+^ secretion (I_SC-amil/insen_), we had yet to characterize the rest of K^+^ pathway on the apical membrane (Fig. 2). The apical application of 5 mM Ba^2+^, a non-specific K^+^ channel inhibitor [26], had no effect on butyrate-stimulated K^+^ secretion in the colonic mucosa. The efficacy of the Ba^2+^ blockade may have been markedly underestimated because binding with mucins and carbonate ions reduces the effective free concentration of Ba^2+^ in microenvironment near apical membrane. This Ba^2+^-insensitive K^+^ conductance was identified as a bupivacaine-sensitive two-pore domain K^+^ channel in H441 human airway epithelial cells [31]. However, bupivacaine did not exert inhibitory effects on butyrate-stimulated I_SC-amil/insen_ in the mucosa in our preliminary experiments. Further studies will be required in order to characterize the electrophysiological and pharmacological properties of butyrate-stimulated K^+^ conductance at single cell level.

In the present study, the application of butyrate to both apical and basolateral solutions shifted I_SC-amil/insen_ in a negative direction (Fig. 1a), indicating an increase in cation secretion or anion absorption and/or a decrease in cation absorption or anion secretion. The Na^+^-K^+^-2Cl^−^ cotransporter is one of the transport systems that includes Na^+^ transport and supplies K^+^ and Cl^−^ from the basolateral membrane in the surface cells of the colonic epithelium [48, 50]. The basolateral application of bumetanide, a Na^+^-K^+^-2Cl^−^ cotransporter inhibitor, inhibited the butyrate-induced I_SC-amil/insen_ shift (Fig. 1c). Among ion transport pathways related to the Na^+^-K^+^-2Cl^−^ cotransporter, an increase in K^+^ secretion matches the direction of the negative shift. On the other hand, a decrease in Cl^−^ secretion on the apical membrane via the cystic fibrosis transmembrane conductance regulator, CFTR, may not be involved in the shift by butyrate. We added 5 μM indomethacin to the basolateral solution to suppress the production of endogenous prostaglandins, which may have increased intracellular cAMP concentrations and, as a result, activated cAMP-activated CFTR Cl^−^ channels. Further experiments with specific inhibitors are required in order to elucidate the pathway of cation secretion in the rat rectal colon.

### Transport pathway for butyrate in rat rectal colon

The relationship between the I_SC-amil/insen_ shift and concentration of butyrate was fit well by the Hill equation (Fig. 4). The value of K_D_, 12.0 mM, corresponds approximately to that of 5.6 mM of the equine large intestine [41]. A K_D_ value for the dependency of pH_i_ acidification on propionate concentrations was reported as 32 mM [12]. Previous studies showed that the concentration of butyrate may reach approximately 10 to 20 mM in rat and other mammalian colons [54], implying that butyrate plays a physiological role in the transport of ions and intracellular factors. In addition, the K_D_ value obtained in the present study was similar to that for the uptake of butyrate into luminal membrane vesicles in pig colon [49] and MCT1 in tumor cells [9]. This similarity supports the I_SC-amil/insen_ shift being due to the absorption of butyrate through MCT1. The Hill coefficient, which was estimated to be 2.9, suggested that butyrate induces K^+^ secretion with positive cooperativity [3]. Previous studies described the transport of monocarboxylates or lactate via MCT1/4, particularly in the brain and muscle [25]. The SCFA^***−***^/H^+^ transport ratio of MCT1 was shown to be 1:1 [4]. Furthermore, the transport of butyrate was found to be increased under acidic conditions [10]. Thus, butyrate may be absorbed by an electroneutral pathway via MCT1, MCT4, and/or MCT5 in the colon [19, 22, 25, 32].

The apical application of 30 mM butyrate caused a markedly smaller I_SC-amil/insen_ shift than its basolateral application (Fig. 5f). Previous studies demonstrated that butyrate markedly changed the intracellular pH of enterocytes from the serosal rather than the mucosal side [8]. Immunohistochemical analyses also showed that MCT1 was predominantly distributed in the basolateral membrane of the surface epithelium and crypts of mice, rats, and humans [19, 32, 53]. In contrast, MCT1 was only weakly detected in the apical membrane of surface cells [53]. Thus, low permeability at the apical membrane serves as the rate-limiting step for the overall absorption of butyrate in colonic epithelial cells.

The negative shift caused by butyrate may also result from a decrease in cation absorption. However, the stoichiometry of SMCT1, which is considered to be 3:1 or 2:1 between Na^+^ and monocarboxylate [13, 14], is expected to induce an increase in cation absorption. Additionally, ibuprofen as an SMCT1 inhibitor [13, 42] did not exert any effects on I_SC_ after the administration of butyrate (Fig. 5c). Thus, a large amount of butyrate was not transported via SMCT1 in rat rectal colon. Kaji et al. [36] recently showed that acetate (maximum concentration of 30 mM) activated I_SC_ in rat duodenum and attributed this to the activation of SMCT1. However, in that study, HCO_3_^−^-free bathing solution was used on the apical side and HCO_3_^−^-containing bathing solution on the basolateral side. Differences in the region of the gastrointestinal tract and/or experimental settings may result in different conclusions.

Previous studies demonstrated that butyrate induced bicarbonate secretion and that SCFA was transported via the SCFA^−^/HCO_3_^−^ exchanger [2, 16]. SLC26A3 was also shown to be preferentially expressed over other SLC26 family members in rat rectal colon [52, 57]. SLC26A3 expressed in *Xenopus* oocytes only weakly transported butyrate [11]. However, no significant differences were observed in the I_SC-amil/insen_ shift between control and HCO_3_^−^-free conditions in rat rectal colon (Fig. 5b). While SLC26A3 and 6 also mediate SCFA^−^/Cl^−^ exchange under HCO_3_^−^-free conditions [40, 51], the SCFA^−^/HCO_3_^−^ exchange may not be the electrogenic transport pathway for butyrate.

Previous studies reported that rat rectal colon (RC), distal colon (DC), and ileum (IL) expressed the mRNAs of MCT1 and 4 [32], as well as those of SMCT1 [32, 42], SLC26A3, and SLC26A6 [46, 52]. In the present study, we confirmed expression at the mRNA level of *Slc16a1* (MCT1), *Slc16a3* (MCT4), and *Slc16a4* (MCT5), which may participate in SCFA transport (Fig. 6). However, we cannot rule out unidentified transporters and non-ionic diffusion via the lipid membrane because butyrate is the most lipophilic SCFA [56].

### Regulation of electrogenic Na^+^ transport via ENaC by butyrate

In our previous study, amiloride-sensitive I_SC_ in rectal colon of rats fed a normal Na^+^ diet was enhanced after a 5- to 8-h incubation with aldosterone [29]. In addition, Zeissig et al. [61] showed that 1.25 mM butyrate increased dexamethasone-activated, electrogenic sodium absorption via ENaC in rat distal colon 8 h after its application. However, our results demonstrated that amiloride-sensitive I_SC_ (I_SC-amil/sen_) was not affected by butyrate at a concentration of 30 mM within 1 h in rat rectal colon (Fig. 1a, b). These results indicated that butyrate regulated the transcription and/or translation of ENaC subunits as well as the insertion of channels into the apical membrane of enterocytes, but not the activity of ENaC in the short term.

### Physiological significance of butyrate in colon

Previous studies demonstrated that butyrate and monocarboxylates decrease the intracellular pH of colonocytes in rodents [8, 17] and MCT1-expressing cells, respectively, without changing the membrane potential [4]. The acidification of cytosolic pH increased whole-cell currents in KCNQ1/KCNE1-expressing cells [55]. In addition, intracellular acidification with butyrate augmented the amplitude of voltage-dependent K^+^ currents in canine pulmonary arterial smooth muscle cells [1]. KCNQ4 channels were shown to regulate the tone of rat pulmonary artery [34]. Thus, KCNQ4 channels may maintain the membrane potential of colonic epithelial cells under intracellular acidification with butyrate.

Butyrate also increased cell volume in rat distal colon [17]. KCNQ1 and KCNQ4 channels were regulated by cell volume changes through the interaction of the N terminus of the channel protein with the cytoskeleton in *Xenopus* oocytes [24]. Previous findings suggested that phosphatidylinositol 4,5-bisphosphate (PIP_2_) was involved in the volume sensitivity of KCNQ1/KCNE1 channels expressed in COS-7 cells [43]. It was hypothesized that the intracellular concentrations of Mg^2+^ and polyamines are diluted during cell swelling, resulting in a weaker interaction between these cations and PIP_2_, leading to channel activation. KCNQ-type K^+^ channels may have been regulated by butyrate via intracellular pH and cell volume changes in colonic cells. However, the metabolism of butyrate, even in the presence of glucose, may lead to the production of substrates, such as ATP, which stimulate K^+^ channels.

The concentration of SCFAs in peripheral blood was one thousandth that in the large intestine, suggesting that SCFAs are actively metabolized both in the gastrointestinal tract and in other organs such as liver and muscles [44]. However, a previous study revealed that the concentration of SCFAs in peripheral blood of patients with acute salmonellosis caused by *Salmonella enteritidis and S. typhimurium* was significantly and consistently higher, from 5- to 20-fold, than that in healthy subjects [39]. Many pathogens interact with elements of the intestinal barrier, which emphasizes the importance of bacterial-host interactions. For example, they have been shown to compromise epithelial tight junctions, resulting in the excessive translation of luminal contents, including butyrate, into the systemic circulation [6]. Although the pathophysiological concentration of butyrate in the basolateral interstitium currently remains unknown, butyrate may be taken into colonic cells via basolateral MCTs from interstitial fluid and metabolized. The results of the present study indicate that KCNQ-type K^+^ channels contribute to intestinal secretion, flushing out enteric bacteria, and maintaining the environment in the lumen.

In conclusion, we herein showed that butyrate regulated K^+^ secretion through KCNQ-type K^+^ channels and may be absorbed by an electroneutral pathway via H^+^-coupled MCTs, such as MCT1, in the apical membrane of rat rectal colon. KCNQ-type K^+^ channels composed of KCNQ2 and KCNQ4 may play a role in intestinal secretion and defense mechanisms in the case of infection and loss of epithelial integrity.

## Abbreviations

CFTR: Cystic fibrosis transmembrane conductance regulator
DC: Distal colon
ENaC: Epithelial Na^+^ channel
IL: Ileum
I_SC_: Short-circuit current
I_SC-amil/insen_: Amiloride-insensitive short-circuit current
I_SC-amil/sen_: Amiloride-sensitive short-circuit current
KRB: Krebs-Ringer bicarbonate solution
MCT: Monocarboxylate transporter
pKa: Logarithmic acid dissociation constant
RC: Rectal colon
SCFA: Short-chain fatty acid
SD rats: Sprague-Dawley rats
SLC: Solute carrier
SMCT1: Na^+^-coupled transporter for monocarboxylates.
